# Hospitals and Dispensaries in Burma

**Published:** 1904-04-30

**Authors:** 


### HOSPITALS AND DISPENSARIES IN BURMA.

On January 1st, 1902, there were 171 hospitals and dispensaries of all classes at work in Burma, which, with regard to the population, was at the rate of one hospital for every 59,195 souls. During the previous year seven new institutions had been opened and one closed, or rather removed to another locality. The total number of in-patients treated was 42,000, against 39,000 in 1901; and the out-patients who sought relief at the dispensaries numbered 883,000, or 25,000 in excess of the figures of the previous year. It is still difficult to convince patients of the necessity of attending personally for treatment. No less than 24 per cent. of the total outdoor attendance was by proxy, and although the civil surgeons are constantly impressing upon the natives the unsatisfactory nature of medical advice given without personal examination, as yet the advice has not borne much fruit. The long and difficult distances which have often to be traversed from house to hospital are, of course, one of the chief causes of this undesirable state of things. The average number of days each patient was under treatment—exclusive of the leper asylums—was 1.9 for the outdoor patients, and 17.8 for the indoor patients. The total number of surgical operations performed during the year was 22,000, against 20,000 in 1901, including 92 major amputations, 64 trephining, 60 extraction of the lens for cataract, and 64 laparotomy. The total of deaths amongst these patients was ·64 per cent. The percentage of deaths of other patients was lower than in the previous year. In the Rangoon General Hospital the average number of beds occupied was 79 per cent., and there was more or less overcrowding in the native wards throughout the year. The number of nurses was 21, and the average number of patients to each nurse 17.3, as compared with 7.36 in the Medical College Hospital, Calcutta. At the Dufferin Hospital eight pupil midwives were trained, and Dufferin Hospital nurses were employed by municipalities at 23 smaller institutions. Amongst other improvements at the Burmese Hospital, mosquito curtains are now in use in all the wards. There is still a difficulty in getting civil hospital assistants for Burmese hospitals and the department during the year was considerably under-manned, the reply to an appeal made to the Indian Medical Schools holding out no hope that assistance could be looked for from the Northern Indian schools. The question of a local medical school is at present under consideration, and though in some quarters it is not regarded as a complete solution of the present difficulty, it seems to be the only measure which will fairly meet the case.

